# Editorial: Noncovalent interactions in N/O heterocycles

**DOI:** 10.3389/fchem.2025.1763239

**Published:** 2025-12-17

**Authors:** Subbiah Thamotharan, M. Judith Percino, Diego Mauricio Gil

**Affiliations:** 1 Biomolecular Crystallography Laboratory and DBT-Bioinformatics Center, School of Chemical and Biotechnology, SASTRA Deemed University, Thanjavur, India; 2 Unidad de Polímeros y Electrónica Orgánica, Instituto de Ciencias, Benemérita Universidad Autónoma de Puebla, Puebla, Mexico; 3 INBIOFAL (CONICET-UNT), Instituto de Química Orgánica, Facultad de Bioquímica, Química y Farmacia, Universidad Nacional de Tucumán, San Miguel de Tucumán, Argentina

**Keywords:** DFT, Hirshfeld surface, molecular docking, N/O heterocycles, weak noncovalent interactions

Noncovalent interactions are often described as the “soft glue” of chemistry, yet their collective influence is anything but weak (Desiraju et al., 2011; Schneider, 2022). In nitrogen- and oxygen-containing heterocycles, these forces range from classical hydrogen bonds to σ-hole contacts (Frontera and Bauzá, 2021; Siddiqui et al., 2024), π-stacking, and dispersion-driven interactions. Together, they play decisive role in controlling structure, molecular recognition, stability, reactivity, and functional behaviour across organic, inorganic, biological and materials systems. This Research Topic, Noncovalent Interactions in N/O Heterocycles, brings together four original contributions that demonstrate how understanding such subtle interactions can offer predictive control over molecular assembly and crystal engineering.

Al-Wahaibi et al. analyze a series of 1,2,4-triazole-3-thione–linked adamantyl derivatives and identify a remarkably diverse set of contacts, including C–H···N, C–H···O, C–H···Cl, C–H···S, π–π stacking, and chalcogen-based σ-hole interactions. Using single-crystal X-ray diffraction together with Hirshfeld surface analysis, Quantum Theory of Atom in Molecules (QTAIM), and generalized Kohn–Sham energy decomposition, they show how conventional and less common interactions work cooperatively to stabilize supramolecular network. Molecular docking with urokinase plasminogen activator suggests that optimizing these interactions at the protein-ligand interface may enhance antiproliferative activity, emphasizing their biological relevance. The authors also highlight the usefulness of HOMO/LUMO-based descriptors in correlating ligand electronic structure with predicted binding affinity.

Guelmami et al. examine 4-dimethylaminopyridinium-dihydrogen monophosphate, an organic–inorganic hybrid stabilized by N–H···O and O–H···O hydrogen bonds connecting the phosphate group to the heterocycle. Through MEP mapping, HOMO–LUMO analysis, ELF/LOL topology, and RDG–NCI surfaces, they show how electrostatic and dispersive contributions define the supramolecular framework. Their work illustrates that protonated N-heterocycles provide versatile building blocks for robust supramolecular architectures, with implications for molecular engineering, materials science, and functional solid-state systems.

Two additional contributions extend these insights to metalloporphyrin systems, which feature nitrogen-rich macrocycles central to many biochemical and catalytic applications. Dhifet et al. report the synthesis and characterization of a hydrogencarbonato iron(II) picket-fence porphyrin, stabilized by N–H···O, C–H···O and C–H···π interactions. Their combined crystallographic and theoretical analysis (DFT, QTAIM, ELF/LOL, and Hirshfeld analyses) reveal how electron distribution and intermolecular complementarity determine crystal packing and magnetic behaviour. Salhi et al. examine a chloride-bound, five-coordinate high-spin Fe(II) picket-fence porphyrin, where C–H···O/N/π contacts together support the supramolecular arrangement. Collectively, these studies emphasize that coordination geometry, electronic structure, and supramolecular order in metalloporphyrins are shaped by a delicate balance of weak interactions.

Across these studies, several shared messages emerge. First, noncovalent interactions are inherently cooperative. Even very weak contacts, such as H···H dispersion in adamantyl groups, can influence overall structure when combined with stronger directional forces. Second, N/O heterocycles remain exceptionally adaptable scaffolds, capable of engaging in charge-assisted hydrogen bonding, chalcogen bonding, π-stacking, and a range of electrostatic interactions. Third, coupling high-quality crystallographic data with modern theoretical methods is now indispensable for quantifying energetics, directionality, and interaction cooperativity. Increasingly, integrative methodologies that combine crystallography, computational chemistry, and structural database mining are driving new advances in supramolecular chemistry, drug discovery, and materials science (Groom et al., 2016; Jelfs, 2022; Spackman and Jayatilaka, 2009).

Importantly, the works in this Research Topic highlight how the study of weak interactions is evolving from simple qualitative descriptions toward predictive design principles. As computational power, theoretical techniques, and machine learning tools continue to grow (Tretiakov et al., 2025), we anticipate accelerated development of rational strategies for constructing functional molecular solids, biomimetic assemblies, and targeted small-molecule therapeutics.

## References

[B1] Desiraju G. R. Vittal J. J. Ramanan A. (2011). Crystal engineering: a textbook. World Sci.

[B2] Frontera A. Bauzá A. (2021). On the importance of σ–Hole interactions in crystal structures. Crystals 11, 1205. 10.3390/cryst11101205

[B3] Groom C. R. Bruno I. J. Lightfoot M. P. Ward S. C. (2016). The Cambridge structural database. Acta Crystallogr. Sect. B 72, 171–179. 10.1107/S2052520616003954 27048719 PMC4822653

[B4] Jelfs K. E. (2022). Computational modeling to assist in the discovery of supramolecular materials. Ann. N. Y. Acad. Sci. 1518, 106–119. 10.1111/nyas.14913 36251351 PMC10091946

[B5] Schneider H.-J. (2022). Noncovalent interactions: a brief account of a long history. J. Phys. Org. Chem. 35, e4340. 10.1002/poc.4340

[B6] Siddiqui R. Rani J. Titi H. M. Patra R. (2024). Unravelling the potential of sigma hole-assisted co-crystallization: highlighting recent developments. Coord. Chem. Rev. 517, 215994. 10.1016/j.ccr.2024.215994

[B7] Spackman M. A. Jayatilaka D. (2009). Hirshfeld surface analysis. CrystEngComm 11, 19–32. 10.1039/B818330A

[B8] Tretiakov S. Nigam A. Pollice R. (2025). Studying noncovalent interactions in molecular systems with machine learning. Chem. Rev. 125, 5776–5829. 10.1021/acs.chemrev.4c00893 40489661 PMC12203479

